# A reductionist approach to determine the effect of cell-cell contact on human epidermal stem cell differentiation

**DOI:** 10.1016/j.actbio.2022.07.054

**Published:** 2022-09-15

**Authors:** Blaise Louis, Mukul Tewary, Andrew W. Bremer, Christina Philippeos, Victor A. Negri, Sebastiaan Zijl, Zev J. Gartner, David V. Schaffer, Fiona M. Watt

**Affiliations:** a Centre for Stem Cells and Regenerative Medicine, King's College London, Guy's Hospital, SE1 9RT, UK; b University of California, Berkeley, 278 Stanley Hall, Berkeley, CA 94720-3220, USA; c Department of Pharmaceutical Chemistry, University of California at San Francisco, 600 16th St, Rm N512E, UCSF Box 2280, San Francisco, CA 94158, USA

**Keywords:** Epidermis, Keratinocytes, Differentiation, Cell-cell contact, Lipid-modified oligonucleotides, Micropatterning

## Abstract

The balance between stem cell renewal and differentiation is determined by the interplay between intrinsic cellular controls and extrinsic factors presented by the microenvironment, or ‘niche’. Previous studies on cultured human epidermis have utilised suspension culture and restricted cell spreading to investigate regulation of differentiation in single keratinocytes. However, keratinocytes are typically adherent to neighbouring cells *in vivo*. We therefore developed experimental models to investigate the combined effects of cell-ECM adhesion and cell-cell contact. We utilized lipid-modified oligonucleotides to form clusters of keratinocytes which were subsequently placed in suspension to induce terminal differentiation. In this experimental model cell-cell contact had no effect on suspension-induced differentiation of keratinocytes. We next developed a high-throughput platform for robust geometrical confinement of keratinocytes to hexagonal ECM-coated islands permitting direct cell-cell contact between single cells. As in the case of circular islands, differentiation was stimulated on the smallest single hexagonal islands. However, the percentage of involucrin-positive cells on small bowtie islands was significantly lower than on single islands, demonstrating that cell-cell contact reduced differentiation in response to decreased substrate adhesion. None of the small bowtie islands contained two involucrin-positive cells. Rather, if one cell was involucrin-positive the other was involucrin-negative. This suggests that there is intrinsic asymmetry in the effect of cell-cell contact in decreasing differentiation. Thus, our reductionist approaches provide new insights into the effect of the niche on keratinocyte differentiation.

**Statement of significance:**

Stem cell behaviour is regulated by a combination of external signals, including the nature of the adhesive substrate and cell-cell interactions. An understanding of how different signals are integrated creates the possibility of developing new biomaterials to promote tissue regeneration and broaden our understanding of skin diseases such as eczema and psoriasis, in which stem cell proliferation and differentiation are perturbed. In this study we have applied two methods to engineer intercellular adhesion of human epidermal stem cells, one involving lipid-modified DNA and the other involving hexagonal micropatterns. We show that the effect of cell-cell adhesion depends on cell-substrate adhesion and uncover evidence that two cells in equivalent environments can nevertheless behave differently.

## Graphical abstract

**Figure:**
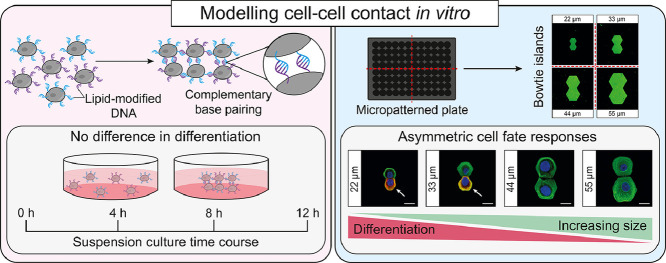

## 1 Introduction

Stem cells harbour the remarkable ability to both self-renew and produce progeny able to differentiate into specialised cell types [1,2]. Understanding the factors that influence stem cell behaviour is vital for the advancement of the field of regenerative medicine. In adult tissues, distinct populations of stem cells are responsible for maintenance and regeneration of the tissue throughout life. The balance between stem cell renewal and differentiation relies on the interplay between intrinsic controls within the cell and extrinsic factors presented to the cell by the microenvironment, termed the ‘niche’ [3]. Key components of the niche include homo- and heterotypic cell-cell contact, cell-extracellular matrix (ECM) adhesion, mechanical stimuli, secreted factors and physiological factors such as oxygen and pH [1,4]. The task of elucidating how these extrinsic factors affect stem cell behaviour calls for *in vitro* cell culture models which allow the exposure of stem cells to defined niche factors in a controlled manner [5].

Mammalian skin is a useful organ for investigating how niche factors affect stem cell behaviour due to the presence of populations of stem cells in defined locations and a rapid rate of cell turnover [1,2]. The epidermis is a stratified epithelium forming the outermost layer of skin and provides protection from the harmful threats of the environment [6]. This layer is made up of the interfollicular epidermis (IFE) and adnexal structures such as the hair follicles, sweat and sebaceous glands, which project into the underlying dermis [6,7]. The IFE is a highly organised tissue comprising multiple layers of epithelial cells. The most prominent cell type is the keratinocyte, accounting for 95% of all cells in the IFE [7].

Homeostatic regulation of the IFE and its associated structures is a complex process that ensures the renewal of the epidermal barrier and depends on tissue resident stem cells [8], [9], [10]. Proliferative cells in the innermost basal layer of the IFE divide to produce cells which detach from the underlying basement membrane and undergo a programme of terminal differentiation, giving rise to the spinous, granular and cornified layers [11]. Upwards migration of these cells from the basal to suprabasal layers is accompanied by a series of biochemical changes, including synthesis of differentiation-related proteins such as involucrin and transglutaminase 1, and morphological changes culminating in the dead, flattened corneocytes of the cornified layer [12].

The ability to culture human epidermal stem cells and present them with niche factors which either support the stem cell compartment or induce terminal differentiation has provided a useful experimental model to investigate stem cell fate [13], [14], [15], [16]. Previous studies have shown that when suspended in a viscous methylcellulose-containing medium, single keratinocytes withdraw from the cell cycle and undergo terminal differentiation [15]. This suspension-induced differentiation can be partially inhibited by addition of fibronectin or anti-integrin antibodies to the medium, highlighting the importance of cell-ECM adhesion in the regulation of differentiation [17]. Transcriptomic and proteomic analysis of keratinocytes harvested from suspension over a 12 h time course has shown that commitment to differentiation occurs at 4 h and is characterised by upregulation of several interacting protein phosphatases, whilst differentiation is initiated at 8 h [18].

Another technique for investigating how ECM adhesion affects keratinocyte differentiation is the use of micropatterned substrates to control the area of ECM-coated substrate available to the cell, which in turn controls cell spreading and shape. Circular adhesive islands which restrict cell spreading inhibit proliferation and induce involucrin expression in keratinocytes [16]. Previous studies have shown that the pro-differentiation stimulus imparted by small ECM-coated islands is due to the rearrangement of the actin cytoskeleton, with the ratio between polymerized F-actin and monomeric G-actin affecting SRF activity [19].

In addition to suspension culture and restricted cell spreading there are a variety of non-genetic techniques to manipulate niche interactions in keratinocytes and other cell types, some of which have applications in tissue engineering and regenerative medicine [20]. In particular, hydrophobic insertion of lipid-modified DNA into the membrane allows rapid and reversible formation of cell-cell contacts and can be applied to a wide array of cell types [21], [22], [23]. Furthermore, micropatterned substrates can be fabricated to capture cell pairs with shared intercellular junctions, providing a reductionist model for investigating cell-cell and cell-ECM interactions, without the added complexity of other niche factors present in the intact tissue [24,25]. More complex cultures, ranging from epidermal aggregates, through cultures incorporating different ECM components, to the generation of human skin from iPS cells, all have great utility for studying stem cell-niche interactions [26], [27], [28], [29]. Nevertheless, with increasing complexity the time during which interactions are established increases from hours through days to weeks. In addition, it becomes harder to explore specific interactions in isolation. The benefit of the reductionist approach of micro-patterned islands is illustrated in studies of the relationship between cell-cell contact area and Notch signalling [30], and the effect of engineered nanomaterials on endothelial cells [31,32]. For endothelial cell pairs, hexagonal patterns were selected to mimic the polygonal shape adopted by confluent cells under physiological conditions [31,32] which is similar to the observed morphology of epidermal cells in whole mounts of intact epidermis and in confluent sheets [33].

Whilst many studies have used suspension culture and restricted cell spreading to investigate stem cell fate in keratinocytes, these cells are very rarely found as single cells *in vivo*. This highlights a need for *in vitro* cell culture models which control cell-ECM adhesion but also allow for direct contact between cells. To investigate the combined effect of cell-ECM adhesion and cell-cell contact we utilized lipid-modified oligonucleotides to form clusters of keratinocytes which were subsequently placed in suspension to induce terminal differentiation. We also developed a high-throughput platform for robust geometrical confinement of keratinocytes to islands permitting direct contact between single cells. This reductionist approach provides insights into the combined effect of cell-ECM adhesion and direct cell-cell contact on keratinocyte differentiation.

## 2 Materials and methods

### 2.1 Preparation of micropatterned plates

96-well micropatterned plates were fabricated using an adaptation of the protocol originally described by Tewary et al. [34,35] for high-throughput screening studies of confined human pluripotent stem cell (hPSC) colonies. Custom Quartz photomasks (JD Photodata) were designed for circular single cell islands (20 and 50 µm diameter), hexagonal single cell islands (areas equivalent to 22, 33, 44 and 55 µm diameter circular islands) and bowtie-shaped islands (two contacting hexagons). The desired patterns were transferred to the surface of coated slides by photo-oxidation of the pattern region through a Quartz photomask by deep UV (DUV) exposure. Substrates were exposed to DUV for 40 min using a UV-Ozone cleaner (Bioforce Nanosciences) and then attached to bottomless 96-well plates using a biocompatible adhesive (ISO 10993 approved). Prior to cell seeding, the wells were activated with N-(3-dimethylaminopropyl)-N′-ethylcarbodiimide hydrochloride (Sigma-Aldrich) and N-hydroxysuccinimide (Sigma-Aldrich) for 30 min. The plates were washed thoroughly with ddH_2_O and incubated with 20 µg/ml rat-tail collagen type I (Corning) in phosphate-buffered saline (PBS, Sigma-Aldrich) overnight at 4ºC. Following incubation, the plates were washed 3 times with PBS to remove passively adsorbed ECM prior to cell seeding. To visualise the patterned regions, the wells were incubated with 20 µg/ml Alexa Fluor 488-conjugated fibrinogen from human plasma (Invitrogen) overnight at 4ºC. The wells were washed thoroughly with PBS prior to imaging.

### 2.2 Cell culture

Frozen primary human keratinocyte stocks (strain Km) previously isolated from surgically discarded human neonatal foreskin [36] were used between passage 3-7 for all experiments. J2-3T3 cells (feeder cells) were originally obtained from Dr James Rheinwald (Department of Dermatology, Harvard Skin Research Centre, USA) and used at passage 4-12. All cells were routinely tested for mycoplasma contamination and were negative. Keratinocytes were cultured in FAD medium (1 part Ham's F12 medium and 3 parts Dulbecco's Modified Eagle Medium (DMEM), Gibco) supplemented with 10^−4^ M adenine, 10% (v/v) fetal bovine serum (FBS, Gibco), 0.5 µg/ml hydrocortisone, 5 µg/ml insulin, 10^−10^ M cholera toxin, 10 ng/ml epidermal growth factor (EGF), 100 IU/ml penicillin and 100 µg/ml streptomycin (Sigma-Aldrich). Feeder cells were maintained in high-glucose DMEM (Gibco) supplemented with 10% (v/v) adult bovine serum (Life Technologies), 100 IU/ml penicillin and 100 µg/ml streptomycin.

Keratinocytes were cultured on a monolayer of mitotically inactivated J2-3T3 cells (2-3 h mitomycin C treatment, 4 µg/ml final concentration, Sigma-Aldrich) in complete FAD medium as described previously [13,36]. For experiments, keratinocytes were harvested a week after seeding (approximately 80% confluence). Following the removal of the feeder layer with Versene (Gibco), keratinocytes were collected by trypsinization (0.05% trypsin in EDTA, Sigma-Aldrich), washed and filtered twice through a 40 µm nylon cell strainer (Falcon) to remove clumps of cells and ensure a single cell suspension. For micropatterned substrate experiments, cells were seeded in complete FAD medium and allowed to adhere for 1 h at 37ºC to select for stem cells [37], before non-adherent cells were washed away using fresh medium. Cells were fixed 2 and 24 h after initial cell seeding.

### 2.3 Immunofluorescence labelling and microscopy

Cells on micropatterned substrates were fixed with 4% paraformaldehyde (PFA, Sigma-Aldrich) for 10 min at room temperature and then permeabilized with 0.2% (v/v) Triton X-100 (Sigma-Aldrich) in PBS for 10 min at room temperature. Fixed samples were incubated in blocking buffer (10% (v/v) goat serum (Sigma-Aldrich) and 0.25% (v/v) cold water fish skin gelatin (Sigma-Aldrich) in PBS) for 1 h at room temperature. Substrates were then incubated in primary antibodies diluted in blocking buffer overnight at 4ºC. Primary antibodies used in this study are listed in Supplementary Table 1. After washing thoroughly with PBS, samples were incubated with Alexa Fluor-conjugated secondary antibodies (Alexa Fluor 488, 594 or 647, Invitrogen) and 4,6′-diamidino-2-phenylindole (DAPI, ThermoFisher Scientific) for 1 h at room temperature, protected from light. Micropatterned plates were then washed with PBS before proceeding to imaging. Images were acquired on an Operetta CLS high-content imaging system (10× objective, PerkinElmer) and an A1R confocal microscope (40× objective, Nikon). Confocal images were processed using Icy software (Quantitative Image Analysis Unit, Institut Pasteur, Paris, France).

### 2.4 High-content imaging analysis

Micropatterned plates were processed for immunofluorescence microscopy as described above and automated image acquisition was performed using the Operetta CLS high-content imaging system (PerkinElmer). Images were analysed using custom analysis pipelines made with the Harmony high-content analysis software package (PerkinElmer). A summarised example of the core imaging analysis pipeline is shown in Supplementary Fig. 1. First, nuclei were identified using the DAPI channel then cells were identified based on immunofluorescence labelling for keratin 14. To ensure that only islands containing one cell were used in the analysis, a clustering step was performed to group cells together based on immediate proximity. Border objects were removed, then islands containing single or multiple cells were identified using linear classifiers based on multiple STAR (Symmetry, Threshold compactness, Axial or Radial) morphology properties and manual image training (PhenoLOGIC machine learning module). A thresholding step for identifying keratin 14-positive cells was performed to ensure no feeder cells were included in the analysis. Cytoplasmic or nuclear ROI were then used to determine the fluorescence intensities of differentiation or proliferation markers respectively. Finally, the background intensity for each field was measured by segmenting the cells from the image background. Cells with intensities above background levels were scored as positive.

### 2.5 Oligonucleotide labelling of cells and cluster formation

Lipid-modified oligonucleotides were obtained from ATDBio (Southampton University). Sequences and lipid modifications for all oligonucleotides used in this study are listed in Supplementary Table 2. Primary human keratinocytes were labelled with lipid-modified DNA according to Weber et al. [23]. Keratinocytes were washed 3 times in calcium and magnesium-free PBS and resuspended at a final volume of 10^6^ cells in 48 µl PBS. Cells were labelled with the single-stranded adhesion DNA by the addition of 1 µl of a 50 µM solution of the anchor strand in water. Cells were gently agitated at room temperature for 10 min before addition of 1 µl of a 50 µM solution of the co-anchor strand in water. Cells were once again gently agitated for 10 min at room temperature followed by three washes in PBS by pelleting and resuspension to remove unbound oligonucleotides.

To quantify cell surface labelling, keratinocytes were incubated with a FAM-conjugated 20mer oligonucleotide with a complementary sequence to the handle portion of the anchor strand. The fluorescent complement was incubated in the dark at 4ºC for 30 min. Cells were washed 3 times in PBS and resuspended in 1% BSA in PBS in preparation for flow cytometry. FITC-conjugated beads from a Quantum MESF bead kit (Bang's Laboratories) were analysed by flow cytometry (Supplementary Fig. 2a) and the median fluorescence intensities were used to generate a calibration plot using the QuickCal v. 2.3 data analysis program (Supplementary Fig. 2b). The median fluorescence intensities of the functionalised cells were then used to determine the MESF value, corresponding to the total number of DNA strands per cell. Data acquisition was performed using the FACSCanto system (BD Biosciences) and data analysis was performed using the FlowJo software package, version 10 (Tree Star, Ashland, OR).

Keratinocytes were surface functionalised with complementary lipid-modified DNA (A or A’). To identify two separate populations, CellTracker Green CMFDA and Red CMTPX dyes (Invitrogen) were used to fluorescently label cells according to the manufacturer's protocol before proceeding through the oligonucleotide labelling steps described above. Green and red cells were mixed at a ratio of 1:1 or 1:4 at a total concentration of 10^6^ cells per 200 µl of ice-cold PBS and allowed to adhere for 30 min before proceeding with flow cytometry.

### 2.6 Suspension-induced differentiation

For suspension-induced differentiation experiments, cells conjugated with complementary lipid-modified DNA were mixed at a ratio of 1:1 to generate clusters. Single and clustered keratinocytes were differentiated in suspension as described previously [17,18]. 6-well tissue culture plates (Corning) were coated with 0.4% polyhydroxyethylmethacrylate then air-dried for 1 h. 1.45% methylcellulose in complete FAD medium was added to each well and 10^6^ cells were mixed into the medium using a pipette tip. Cells were incubated at 37ºC and harvested at 4 h time points by diluting the methylcellulose with PBS. Cells were collected and subjected to multiple PBS washes by centrifugation.

### 2.7 Clonogenicity assays

For clonogenicity assays, mitomycin C treated feeder cells were plated in 6-well tissue culture plates. Cell clusters were separated using DNase as described previously [21] to ensure a single cell suspension. 1000 keratinocytes were plated per well (one plate per condition; therefore 6 technical replicates) and cultured for 12 days. Feeder cells were removed by thoroughly washing with PBS and keratinocyte colonies were fixed with PFA for 10 min. Colonies were stained with 1% rhodanile blue (1:1 mixture of rhodamine B and Nile blue A, Acros Organics) for 30 min, washed thoroughly with PBS and then imaged using a Molecular Imager Gel Doc XR System (Bio-Rad Laboratories). ImageJ processing software (National Institutes of Health) was used for automated analysis of the total number of colonies per well and colony area by using the ‘Analyze Particles’ tool, with a minimum particle size of 0.01 mm^2^.

### 2.8 RNA extraction, complementary DNA preparation and quantitative real-time PCR

Total RNA was isolated from keratinocytes using the RNeasy kit (Qiagen) and complementary DNA was reverse transcribed using the QuantiTect Reverse Transcription kit (Qiagen) according to the manufacturer's instructions. Quantitative real-time PCR (qPCR) of cDNA was performed using qPCR primers and Fast SYBR green Master mix (Life Technologies). qPCR primers used in this study are listed in Supplementary Table 3. qPCRs were run on the CFX384 Real-Time System (Bio-Rad). 18S and TBP were used as housekeeping genes for normalization for all qPCRs.

### 2.9 Graphing and statistical analysis

Graphs were generated and statistical analysis was performed using Prism 9 software (GraphPad). For each micropatterned plate, a minimum of four wells (each with nine imaged fields) per island size was analysed for all experiments. For differentiation assays on single circular and hexagonal micropatterned islands, at least 300 cells were analysed for each island size per experiment. For bowtie-shaped hexagonal islands, a minimum of 200 cells (100 bowties) was analysed for each island size per experiment. Quantitative data are presented as mean ± SD or SEM. One-way and two-way ANOVA with appropriate post-hoc tests and two-tailed unpaired t-tests were performed to determine significance. The specific analysis used for each experiment and the number of independent experiments is presented in the figure captions.

## 3 Results

### 3.1 DNA-programmed assembly of intercellular adhesions

Previous studies have reported modification of the cell surface to present short oligonucleotides allowing for ‘velcro’-like attachment to substrates and other cells functionalised with complementary sequences [21]. This method for programmed cell adhesion is ideal for rapid generation of multicellular structures as it does not require genetic manipulation and can be controlled based on oligonucleotide sequence. Subsequent studies have generated oligonucleotides with fatty acid modifications at the 5’ end which passively incorporate into cell membranes due to the hydrophobic nature of the lipid [22,23,38,39]. We used this approach to functionalise keratinocytes with adhesive properties allowing for rapid generation of cell clusters.

**Fig. 1:**
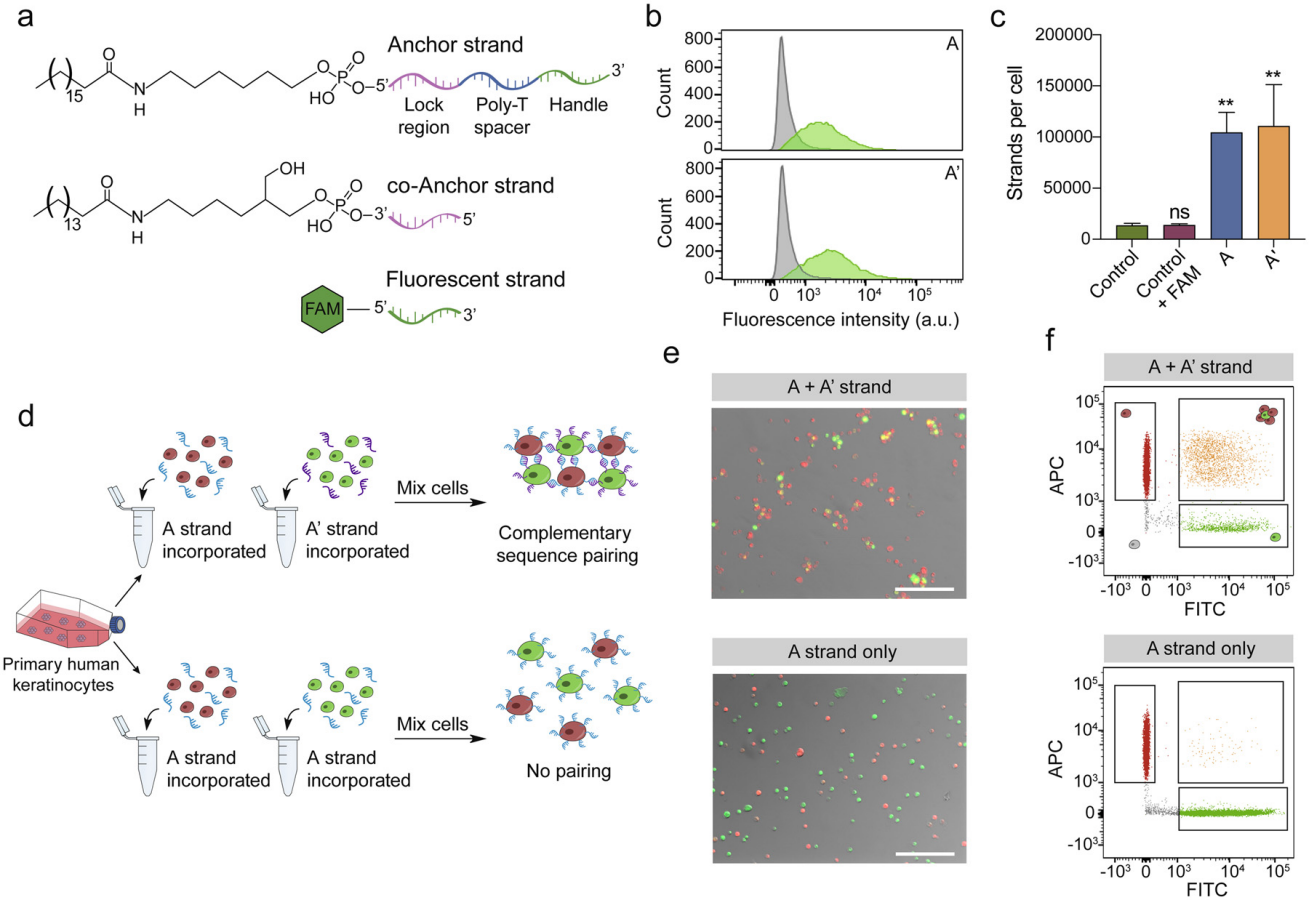
Lipid-modified oligonucleotides integrate into the membrane and impart adhesive properties to cells. (**a**) Structure of the anchor, co-anchor and fluorescent strands. Hybridization between the anchor and co-anchor strand occurs in the lock region, which is followed by a poly-thymine spacer region. The handle region allows for adhesion to other surfaces or complementary fluorescent strands as a means of quantifying membrane incorporation. (**b**) Representative image of flow cytometry analysis of the cell surface after incubating DNA-conjugated cells with the 5’-FAM-modified strand. Similar results were obtained in two replicate experiments. (**c**) Quantification of the number of DNA strands per cell determined by flow cytometry analysis of cells labelled with the 5’-FAM-modified strand and fluorescent beads (see Supplementary Fig. 2 for bead analysis). Data shown are from *n* = 3 independent experiments. Bars represent mean ± SD. *p*-values calculated using one-way ANOVA with Dunnett's multiple comparisons test. **p < 0.01; ns = not significant (*p* > 0.05) compared to the control. (**d**) Schematic describing the method for DNA incorporation and subsequent cluster formation. (**e**) Cells bearing complementary sequences (top) form small clusters when mixed at a ratio of 1:4 (green:red), whilst cells bearing only the A strand do not form clusters (bottom) even when mixed at a ratio of 1:1. Scale bar, 200 µm. (**f**) Representative flow cytometry images of cells bearing complementary sequences mixed at a ratio of 1:4 (top), or cells labelled with the A strand only (bottom). A double-positive population of clustered red and green cells is only seen with complementary DNA (top).

To quantify the extent of lipid-modified oligonucleotide labelling of keratinocytes we incorporated the anchor and co-anchor strands into the cell membrane, followed by a FAM-modified strand complementary to the handle region (Fig 1a). Flow cytometry analysis of labelled keratinocytes established the presence of both A and A’ strands on the cell surface (Fig. 1b). Fluorescently labelled beads were used to establish the total number of oligonucleotide strands per cell to be approximately 104,000 and 110,500 for A and A’ respectively (Fig. 1c, Supplementary Fig. 2a, b). Previous studies have reported similar levels of membrane labelling to be suitable for DNA-programmed cell adhesion [39].

To determine whether the membrane tethered oligonucleotides could facilitate the formation of cell-cell contacts in keratinocytes, we first labelled the cells with cytosolic fluorescent dyes to distinguish between populations. Red and green cells were then labelled with either the A or A’ strand (complementary sequence pairing) or the A strand only (no pairing) (Fig. 1d). The ability of the cells to form large clusters is limited by the availability of neighbouring cells bearing a complementary sequence. When mixed at a ratio of 1:50 the resulting cell clusters are smaller than if mixed at a 1:1 ratio [21]. We mixed cells with complementary sequences at a ratio of 1:4 (green:red) or 1:1 for the control, and observed clustering only between red and green cells with complementary sequences functionalised to the cell surface (Fig. 1e). The clustering of red and green cells was confirmed by flow cytometry analysis (Fig. 1f). We observed a double-positive population of cells labelled with the A and A’ strand, indicating that red and green cells had formed multicellular aggregates. Even when mixed at a ratio of 1:1, cells labelled with only the A strand did not form cell clusters. Combined, these experiments demonstrate that lipid-modified DNA can be functionalised to the surface of keratinocytes allowing the formation of multicellular aggregates through complementary DNA hybridization.

### 3.2 DNA-mediated cell-cell contact does not influence suspension-induced differentiation of keratinocytes

**Fig. 2:**
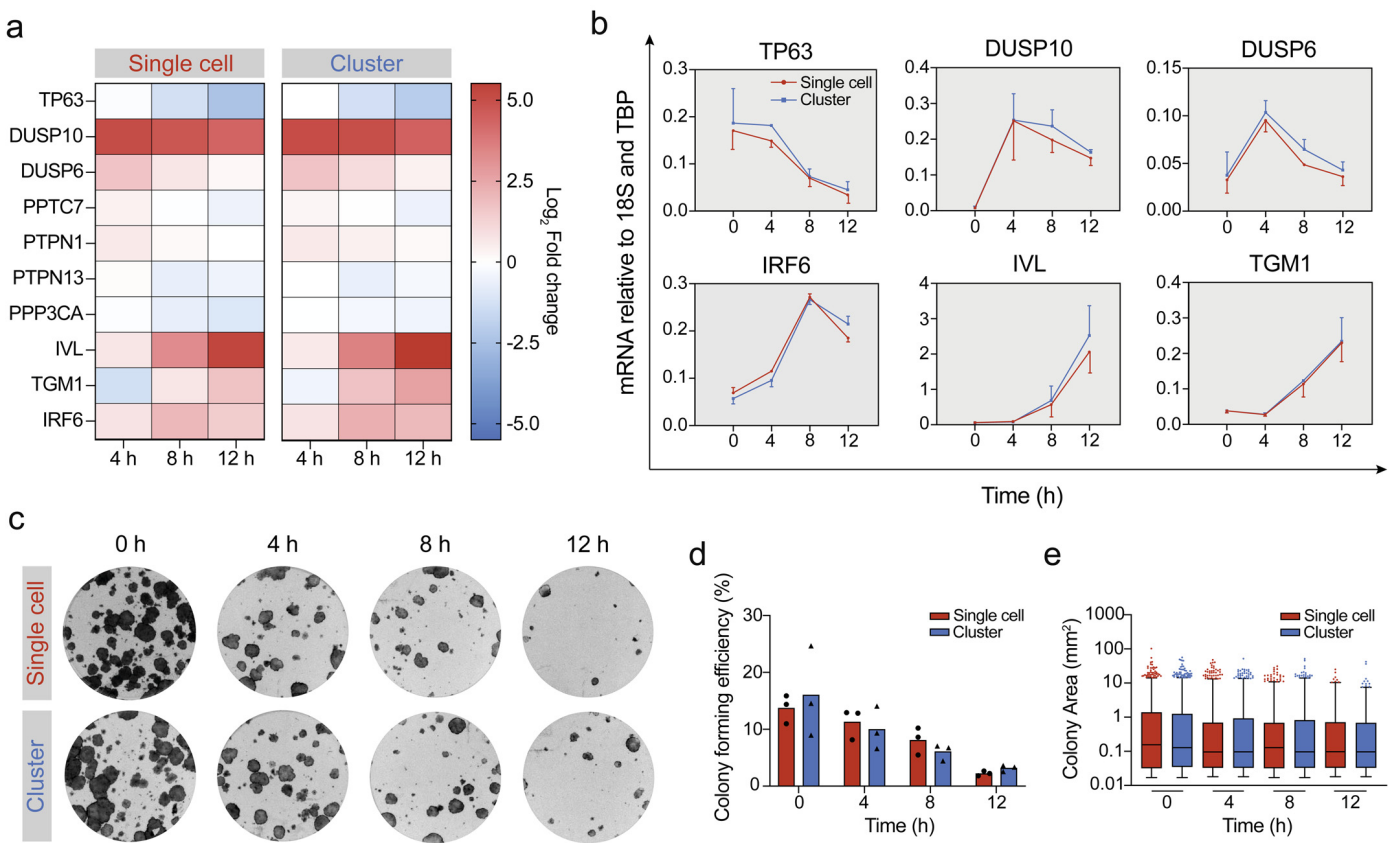
DNA-mediated cell-cell contact does not affect suspension-induced differentiation of keratinocytes. (**a**) Heatmap showing Log_2_ fold change of normalised gene expression during suspension culture of clustered cells at 4, 8 and 12 h relative to 0 h. The mean of *n* = 3 independent experiments was used for each gene except for TP63, IRF6 and TGM1, for which the mean of *n* = 2 independent experiments was used. (**b**) qPCR quantification of TP63, DUSP10, DUSP6, IRF6, IVL and TGM1 mRNA levels relative to 18S and TBP for single cells (red) and clustered cells (blue). Data points represent the mean of *n* = 3 independent experiments for DUSP10, DUSP6 and IVL, and the mean of *n* = 2 independent experiments for TP63, IRF6 and TGM1. Error bars represent SD. (**c**) Representative images showing the effect of suspension-induced differentiation on clonal growth of single and clustered cells after 0, 4, 8 and 12 h. (**d**) Colony-forming efficiency over the suspension time course for single and clustered cells. Data are from *n* = 3 independent experiments with six technical replicates per sample for each time point. Individual points represent average colony-forming efficiency for each experiment; bars represent the mean. (**e**) Quantification of the area of colonies formed by cells harvested from each time point for single and clustered cells (following disaggregation with DNase). Box and whisker plots indicate the median (middle line in the box), 2.5 - 97.5^th^ percentile (bottom and top line on the box respectively) and the minimum and maximum (whiskers).

By functionalising the cell surface with short oligonucleotides we can impart specific adhesive properties to cells. We utilised this approach to rapidly cluster keratinocytes before culturing them in suspension to induce differentiation. Previous studies have shown that keratinocytes commit to differentiation at 4 h in suspension and differentiation occurs at 8 h [18]; we therefore harvested keratinocytes at four-hour timepoints for qPCR analysis. Cells collected immediately after cluster formation served as the 0 h control (Fig. 2a). In both single and clustered cell suspensions expression of the stem cell marker TP63 declined over the time course, whereas terminal differentiation markers involucrin and transglutaminase 1 were strongly upregulated from 8 h. To determine whether cell-cell contact affected commitment, we looked at the expression of protein phosphatases which have previously been shown to peak in expression at 4 h in suspension and promote keratinocyte differentiation [18]. Expression of DUSP10 increased at 4 h andremained high over the time course. DUSP6 and PTPN1 expression increased from 0 to 4 h then declined, while PPTC7, PTPN13 and PPP3CA were all downregulated in suspension. Expression of the Notch signalling target gene IRF6 increased up to 8 h before being slightly downregulated. As in the case of TP63, involucrin and transglutaminase 1, there was no difference in expression of the pro-commitment phosphatases or IRF6 between single and clustered cells (Fig. 2b, Supplementary Fig. 3).

After disaggregating keratinocyte clusters into single cells using DNAse, we performed a clonogenicity assay to determine colony-forming ability. For both single cells and cells from clusters, the number of colonies per well decreased at each time-point (Fig. 2c). We observed no significant difference in colony-forming efficiency when comparing single cells and cells dissociated from clusters (Fig. 2d). We measured the colonies formed for both conditions and found no difference in colony area. These findings indicate that DNA-mediated cell-cell contact has no effect on suspension-induced differentiation of keratinocytes.

### 3.3 A high-throughput platform for investigating shape-induced differentiation in keratinocytes

**Fig. 3:**
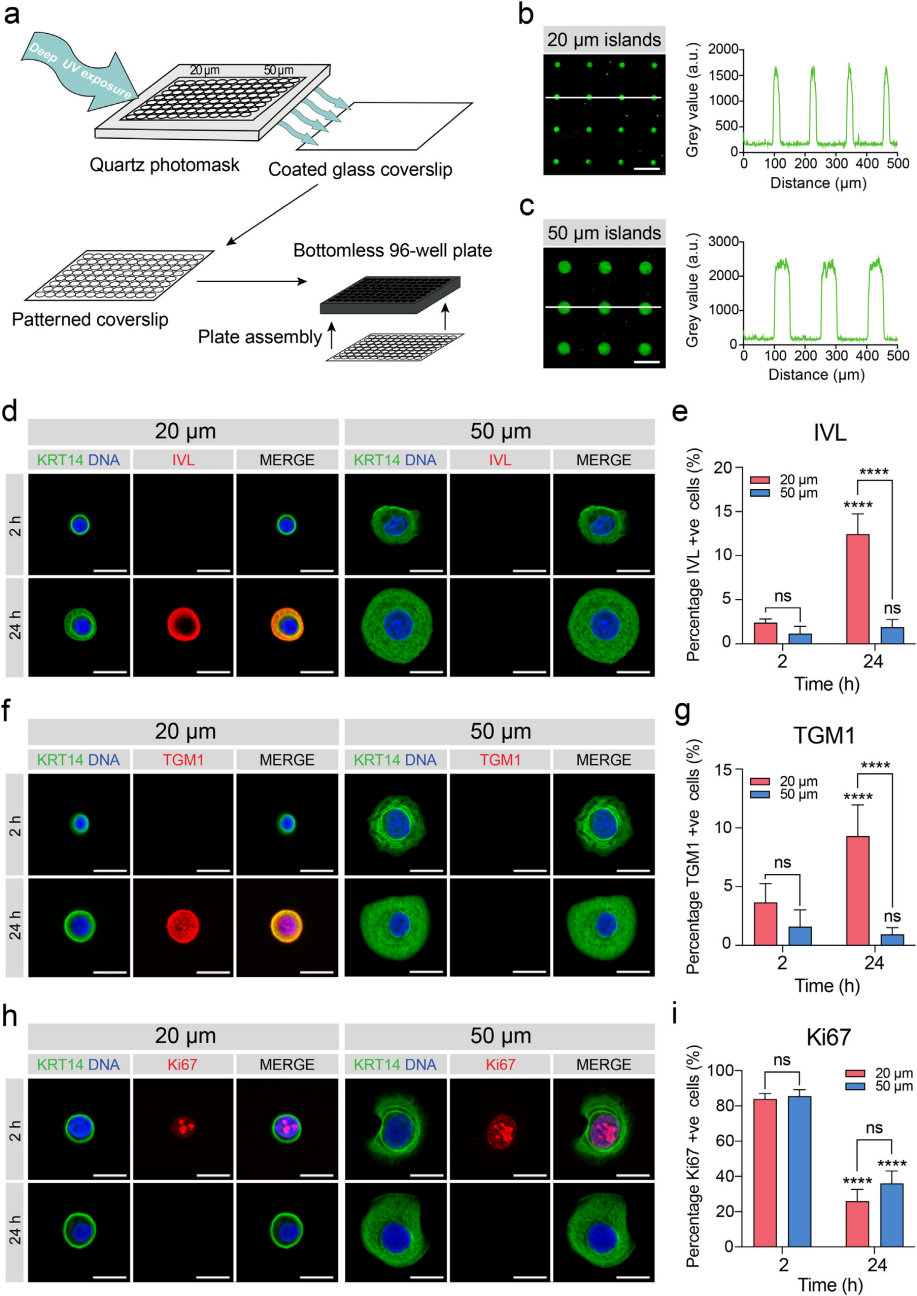
A high-throughput micropatterned platform for shape-induced differentiation of keratinocytes. (**a**) Schematic outlining the method of micropatterned plate fabrication. (**b, c**) Fluorescence microscopy images and intensity profiles of (**b**) 20 and (**c**) 50 µm diameter islands, coated with Alexa Fluor 488-conjugated fibrinogen. Fluorescence intensity profiles correspond to white lines on each image and show uniformity of both pattern shape and distance between islands. Scale bar, 100 µm. (**d, f, h**) Representative immunofluorescence images of keratinocytes on 20 and 50 µm diameter islands at 2 and 24 h after seeding. Cells were labelled for keratin 14 (KRT14, green) and (**d**) involucrin (IVL, red), (**f**) transglutaminase 1 (TGM1, red) and (**h**) Ki67 (red) with DAPI (blue) as a nuclear counterstain. Scale bars, 20 µm. (**e, g, i**) Quantification of the percentage of (**e**) involucrin-positive cells, (**g**) transglutaminase 1-positive cells and (**i**) Ki67-positive cells. Data shown are from *n* = 3 independent experiments. Bars represent mean ± SD. ****p < 0.0001 and ns = not significant (*p* > 0.05) as determined by two-way ANOVA with Šidák's multiple comparisons test.

Previous studies utilising micropatterned substrates have shown that keratinocytes with limited ECM adhesion on 20 µm diameter islands differentiate at a higher frequency than cells on 50 µm diameter islands [19]. The substrates used were fabricated using a micro-contact printing approach to generate a substrate background resistant to protein adsorption. This requires a manual stamping step of the reaction initiator onto gold-coated coverslips which may result in variability between substrates. We aimed to optimise a protocol previously used for geometric confinement of hPSC colonies [35] by scaling it down to the single-cell level, allowing for patterning of single keratinocytes and precise control of cell-cell interactions (Fig. 3a). We designed a Quartz photomask bearing circular islands of 20 and 50 µm diameter. Following photopatterning and plate assembly, carboxyl groups in the DUV exposed regions were activated using carbodiimide and succinimide chemistry to allow for covalent attachment of ECM. We observed robust patterning of both island sizes with consistent distance between neighbouring islands (Fig. 3b, c).

Micropatterned plates were processed for immunofluorescence microscopy. Automated image acquisition was then performed and images were analysed using custom analysis pipelines (Supplementary Fig. 1). To determine whether the circular micropatterned plates direct keratinocyte differentiation, we first selected stem cells by seeding keratinocytes onto both island sizes and then washing off non-adherent cells after 1 h [37]. We determined the percentage of cells which expressed involucrin protein at 2 and 24 h after initial cell seeding (Fig. 3d, e). Keratinocytes were negative for involucrin on both island sizes at 2 h. We observed a significant increase in the percentage of involucrin-positive cells on the 20 µm islands but not on the 50 µm islands after 24 h. Furthermore, the percentage of involucrin-positive cells on 20 µm islands was significantly higher than on 50 µm islands at 24 h. This was also the case for the differentiation marker transglutaminase 1 (Fig. 3f, g). As the onset of differentiation in keratinocytes correlates with withdrawal from the cell cycle, we examined the effect of island size on proliferation by determining the percentage of Ki67-positive cells (Fig. 3h, i). There was a decrease in Ki67 expression after 24 h for both island sizes, but the difference between 20 and 50 µm islands at 24 h was not significant. Taken together, these results confirm earlier findings [19] and demonstrate that we have successfully developed a 96-well micropatterned platform allowing robust geometrical confinement of single cells. By restricting adhesive area on these substrates, we can induce terminal differentiation in keratinocytes.

### 3.4 The combined effects of cell-cell contact and ECM adhesion on keratinocyte differentiation

We next sought to utilise the micropatterned platform to investigate the combined effects of cell-cell contact and ECM adhesion on keratinocyte differentiation. An advantage of the platform is that a variety of island shapes and sizes can be patterned in different wells within the same plate, allowing for high-throughput screening. We designed a photomask with single hexagonal and bowtie-shaped hexagonal islands in four sizes (Supplementary Fig. 4). The areas of each hexagon are equivalent to the area of circular islands with diameters of 22, 33, 44 and 55 µm. Each hexagon of the bowtie provides a constant ECM-coated area to each single cell in the pair and there is direct contact between the two cells along the adjoining edge of the hexagons. As a control we patterned islands with a small gap between each hexagon to prevent contact between the cells.

**Fig. 4:**
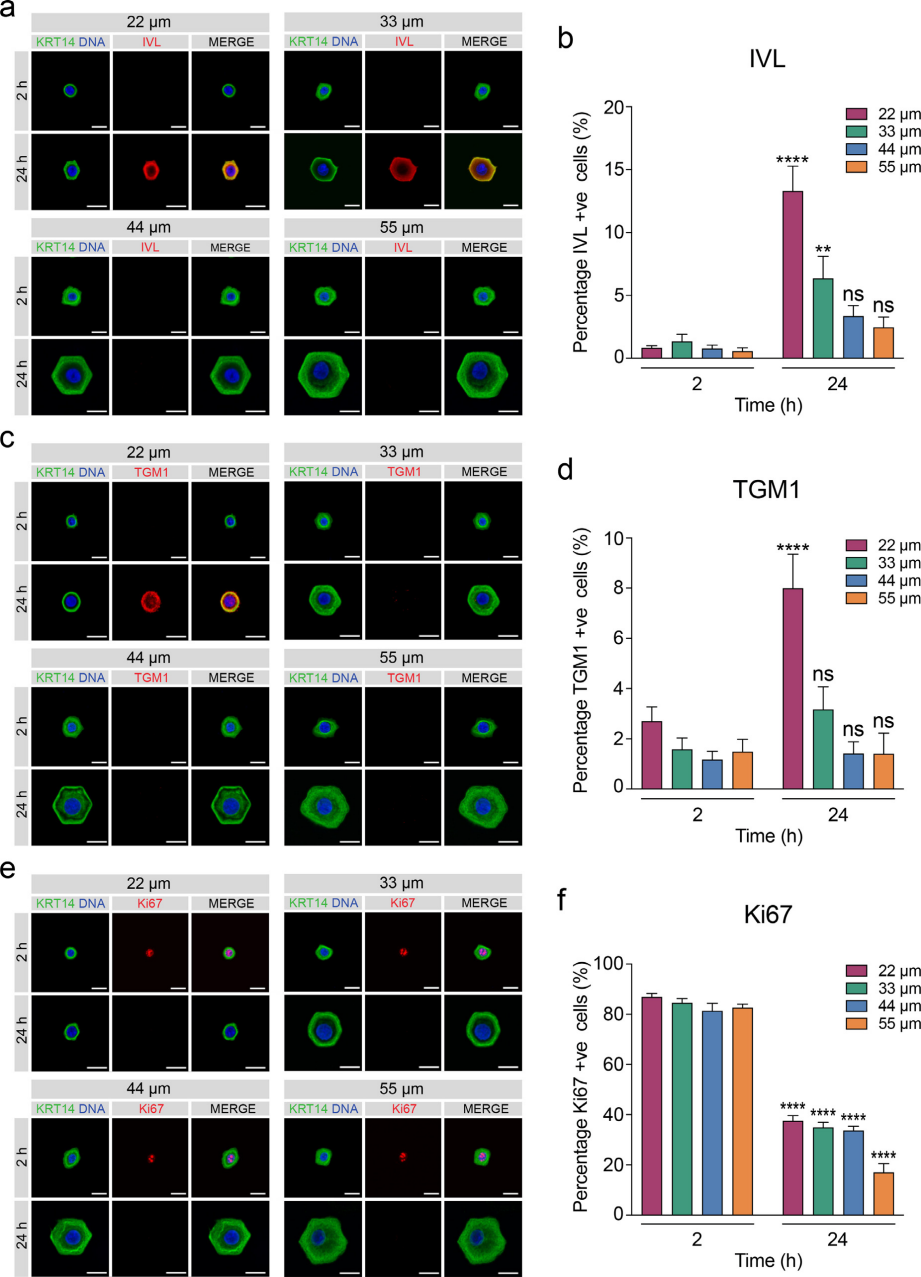
Hexagonal-shaped micropatterned substrates influence differentiation and proliferation of keratinocytes. (**a, c, e**) Representative immunofluorescence images of keratinocytes on single hexagonal islands at 2 and 24 h after seeding. Cells were labelled for keratin 14 (KRT14, green) and (**a**) involucrin (IVL, red), (**c**) transglutaminase 1 (TGM1, red) and (**e**) Ki67 (red) with DAPI (blue) as a nuclear counterstain. Scale bars, 20 µm. (**b, d, f**) Quantification of the percentage of (**b**) involucrin-positive cells, (**d**) transglutaminase 1-positive cells, and (**f**) Ki67-positive cells. Data shown are from *n* = 3 independent experiments. Bars represent mean ± SEM. ****p < 0.0001; **p < 0.01 and ns = not significant (*p* > 0.05) as determined by two-way ANOVA with Šidák's multiple comparisons test.

To test whether single hexagonal islands induce the same differentiation response in keratinocytes as circular islands we quantified the percentage of cells expressing involucrin, transglutaminase 1 and Ki67 at 2 and 24 h after seeding. Keratinocytes were negative for involucrin on all island sizes at 2 h (Fig. 4a, b). A significant proportion of cells were positive for involucrin after 24 h on the two smaller island sizes but not on the two larger-sized islands (Fig. 4b). Keratinocytes were also negative for transglutaminase 1 at 2 h, with the only significant increase at 24 h seen on the smallest island size (Fig. 4c, d). We observed a decrease in the proportion of proliferating cells across all island sizes at 24 h (Fig. 4e, f). These findings indicate that hexagonal-shaped adhesive islands induce similar differentiation responses in keratinocytes to circular islands.

To determine the effect of cell-cell contact on shape-induced differentiation, we performed immunostaining of cell pairs on bowtie-shaped micropatterned islands. We performed immunofluorescence labelling for E-cadherin, the adhesive transmembrane protein component of adherens junctions that associates intracellularly with the F-actin cytoskeleton [40] (Supplementary Fig. 5). We observed the most prominent localisation of E-cadherin at cell junctions on the two smallest island sizes; this was less pronounced on the larger islands. We speculate that cell pairs on the smaller islands may form more robust intercellular adhesions as the smaller bowties provide the most restricted adhesive areas and may therefore limit cell motility.

**Fig. 5:**
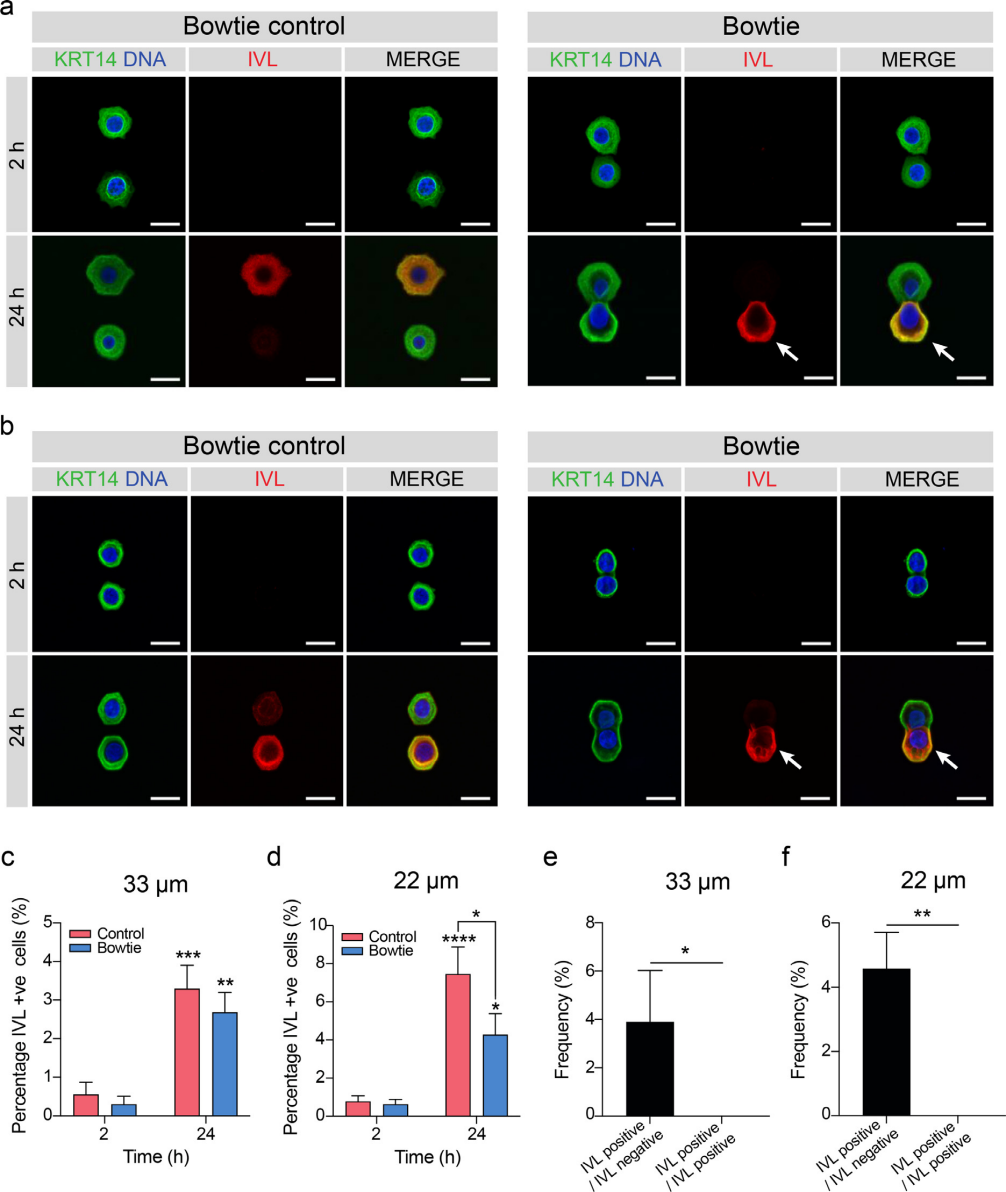
Cell-cell contact reduces involucrin expression in response to decreased substrate adhesion. (**a, b**) Representative immunofluorescence images of keratinocytes on (**a**) 33 µm and (**b**) 22 µm bowtie-shaped islands at 2 and 24 h after seeding. Cells were labelled for keratin 14 (KRT14, green) and involucrin (IVL, red), with DAPI (blue) as a nuclear counterstain. White arrows indicate the involucrin-positive cell present in a cell pair. Scale bars, 20 µm. (**c, d**) Quantification of the percentage of involucrin-positive cells on (**c**) 33 µm bowtie-shaped islands and (**d**) 22 µm bowtie-shaped islands. Data shown are from *n* = 4 independent experiments. Bars represent mean ± SEM. ****p < 0.0001; ***p < 0.001; **p < 0.01; *p < 0.05 and ns = not significant (*p* > 0.05) as determined by two-way ANOVA with Šidák's multiple comparisons test. (**e, f**) Quantification of the frequency of involucrin-positive cells per bowtie on (**e**) 33 µm bowtie-shaped islands and (**f**) 22 µm bowtie-shaped islands. Data shown are from *n* = 3 independent experiments. Bars represent mean ± SD. *p < 0.05, **p < 0.01 as determined by a two-tailed unpaired t-test.

We quantified the expression of involucrin, transglutaminase 1 and Ki67 in micropatterned pairs in which each cell was positioned to opposing sides of the bowtie and made contact along the adjoining edge of the hexagons. Cells were negative for involucrin at 2 h on both the control and bowtie islands of all sizes (Fig. 5a-d, Supplementary Fig. 6a-d). We did not observe a significant increase in involucrin-positive cells or transglutaminase 1-positive cells on both larger sizes of the control and bowtie islands (Supplementary Fig. 7a-d; Supplementary Fig. 6e-h). The percentage of Ki67-positive cells decreased uniformly across all island sizes by 24 h (Supplementary Fig. 8a-h). Interestingly, the percentage of involucrin and transglutaminase 1-positive cells on the bowtie control at 24 h was lower than that observed on single hexagons (Fig. 4a-d). We speculate that is because of the distance between adjacent hexagons. In the case of single hexagonal islands, the distance between each island was 100 µm, whereas the distance between each hexagon in the bowtie control was much smaller, equivalent to the length of the hexagon edge (Supplementary Fig. 4). This reduced distance between single keratinocytes may allow indirect communication between cells mediated by secreted factors [1].

We observed a significant increase in the percentage of involucrin-positive cells on the smallest island sizes (22 µm and 33 µm) after 24 h for both the control hexagons and the bowties (Fig. 5b, d). However, the percentage of involucrin-positive cells on the 22 µm bowtie islands was significantly lower than the control, suggesting that cell-cell contact was affecting differentiation on the smallest island size (Fig. 5d). Although the percentage of transglutaminase 1-positive cells increased for both the bowtie and controls on the smallest island size, this increase was not significant (Supplementary Fig. 7a, b).

We next quantified the percentage of bowties which contained an involucrin-positive and an involucrin-negative cell or two involucrin-positive cells on the smallest island sizes (Fig. 5e, f). None of the islands contained two involucrin-positive cells. Rather, in every case one of the cells was involucrin-positive and one was involucrin-negative (white arrows, Fig 5a, b). This suggests that there is some intrinsic asymmetry in the effect of cell-cell contact in reducing differentiation in our assay conditions, although after 14 days in culture on a feeder layer colonies comprising pairs of involucrin-positive cells can be observed [37].

## 4 Discussion

Cultured human keratinocytes provide a useful experimental model for investigating how stem cell niche factors regulate cell fate and functionality. We have previously highlighted the importance of cell-ECM adhesion in the regulation of terminal differentiation of single keratinocytes by utilising suspension culture and restricting cell spreading on micropatterned islands [16], [17], [18], [19]. However, when several epidermal stem cells are seeded on large collagen-coated islands they assemble into a stratified microepidermis [41], prompting us to investigate the combined effects of cell-ECM adhesion and cell-cell contact in inducing terminal differentiation. To achieve this, we used two different approaches: lipid-modified oligonucleotides and bowtie-shaped micropatterned islands. The duration of the assays was limited to a maximum of 24 h because previous studies have shown that stem cells commit to differentiation at 4 h in suspension and begin to upregulate differentiation markers by 8 h [18]. We did not harvest keratinocytes from the micropatterns and replate them on feeders to measure subsequent colony formation as a readout of stem cell maintenance.

Hydrophobic insertion of lipid-modified DNA into the cell membrane imparted specific adhesive properties to keratinocytes. By mixing cells bearing complementary sequences we formed clusters of keratinocytes which we cultured in suspension. Over the 12 h time course we observed no difference in expression of stem cell, commitment or differentiation markers or in colony forming efficiency when comparing single and clustered cells. We conclude that DNA-mediated cell-cell contact had no effect on suspension-induced differentiation.

We also fabricated micropatterned plates with hexagonal bowtie-shaped islands, which provided a consistent spread area for single cells while also allowing cell-cell contact along the adjoining edge of each hexagon. In this model, cell-cell contact did not induce differentiation on the larger sizes. However, cell-cell contact did reduce differentiation in response to restricted substrate adhesion. Furthermore, we found that none of the cell pairs on the smaller islands contained two involucrin-positive cells after 24 h, which suggests an intrinsic asymmetry in the effect of cell-cell contact in reducing differentiation. In a previous study, keratinocytes were seeded on micropatterned trapezoids and cell-cell contact engineered by placing two trapezoids in contact [42]. In that model cell-cell contact increased differentiation [42], suggesting that the shape of the substrate influences the effect of cell-cell contact.

Microtissues with defined three-dimensional structure and incorporating multiple cell types have been reconstituted *in vitro* through DNA-programmed assembly of cells [38]. By functionalising substrates with NH_2_-terminated oligonucleotides, multiple cell types can be precisely patterned based on the sequence of the oligonucleotide strand on the membrane. This has previously been used to investigate adult neural stem cell fate decisions in response to competing juxtacrine signals from niche cells and immobilised ligands [39,43]. Employing a similar DNA-based patterning approach would be beneficial for investigating how cell-cell interactions between epidermal stem cells and other epidermal niche resident cells influence stem cell fate decisions.

Engineered microenvironments have provided many insights into the extracellular cues that influence stem cell fate [44], [45], [46], [47], [48]. Micropatterned substrates precisely control the adhesive area available to cells by limiting ECM to defined islands surrounded by a protein resistant background. Previously, the extreme protein resistance of poly(oligo(ethylene glycol methacrylate)) brushes has been exploited to achieve high fidelity patterning of single cells [19,49]. This approach relies on soft-lithography and micro-contact printing, requiring a manual stamping step, which prevents large scale production and has the potential to introduce variability between patterned substrates. In contrast, DUV lithography ensures robust pattern fidelity between experiments and allows assembly of patterned microtiter plates for high-throughput screening studies [35,50]. We fabricated 96-well plates with this micropatterning technique and observed a similar differentiation response in single keratinocytes to that seen using micro-contact printing-based fabrication methods [16,19]. Our platform allowed for high-throughput quantification of differentiation of neonatal keratinocytes in response to extrinsic niche factors. By conducting similar studies with adult keratinocytes, the micropatterns could be used to explore age-related niche responsiveness.

We observed accumulation of E-cadherin at cell junctions in micropatterned pairs. Previously, E-cadherin has been implicated as inducing differentiation in micropatterned keratinocyte pairs, as functional blocking of E-cadherin with antibodies or expression of a dominant negative cadherin mutant significantly decreased the percentage of involucrin-positive cells [42]. This is in contrast to the observation that a dominant negative E-cadherin mutant stimulates terminal differentiation of single human keratinocytes in suspension via an effect on beta-catenin signalling [51,52]. Our micropatterned platform will facilitate further studies investigating cell-cell junction assembly and will elucidate the role of E-cadherin and other junctional proteins in differentiation of cell pairs.

We observed asymmetric cell fate decisions on the smallest bowtie islands, whereby only one of the cells underwent differentiation. This leads us to speculate about which intrinsic mechanisms may be involved. Notch signalling is a likely candidate because ligand-dependent Notch activation occurs between cells that are in direct contact and regulates terminal differentiation in the IFE [53]. Stem cell clusters in the basal layer of the epidermis express high levels of the Notch ligand Delta-like 1 (Dll1), which imparts a protective effect on stem cells by blocking Notch signalling, enhances cohesiveness of clusters and induces differentiation in neighbouring cells that express lower levels of Dll1 [54]. Dll1 knockdown in keratinocytes results in increased expression of the Notch target gene IRF6, upregulation of differentiation markers IVL and TGM1 and reduced colony forming ability [55]. In addition, the differentiation stimulus imparted to keratinocytes by substrates functionalised with the Notch ligands Jagged1 and Jagged2 is prevented in cells overexpressing Dll1 [55]. These findings suggest that Dll1 plays a *cis*-inhibitory role by preventing ligand-stimulation of Notch receptors on the cell surface by neighbouring cells [55,56]. Varying levels of Dll1 on the membrane of cells on bowtie islands and the *cis*-inhibitory role played by Dll1 may influence the fate acquired by individual cells. It will be interesting to discover whether the asymmetry of cell fate observed on the bowtie micropatterns mimics asymmetry in epidermal microniches *in vivo*, for example associated with undulations of the epidermal-dermal junction [18,33].

## 5 Conclusions

In our study, we investigated the combined effects of cell-ECM adhesion and cell-cell contact on epidermal stem cell differentiation using reductionist cell culture models. Lipid-DNA mediated cell-cell contact had no effect on suspension-induced differentiation. Our micropatterned platform allowed for high-throughput quantification of differentiation in response to restricted cell spreading. Contact between single cells on bowtie-shaped islands reduced the proportion of involucrin-positive cells relative to control heaxagons. None of the bowties contained two involucrin-positive cells after 24 h, suggesting an intrinsic asymmetry in the effect of cell-cell contact in reducing differentiation. Further studies are necessary to characterise the intercellular signalling events and elucidate the role that cell-cell communication plays in stem cell fate decisions. Our micropatterned platform can facilitate high-throughput studies of stem cell behaviour in response to other extrinsic signals in the epidermal microenvironment, such as heterotypic cell interactions, secreted factors and physiological factors including oxygen and pH levels. Increased understanding of how these factors influence stem cell proliferation and differentiation can potentially lead to new discoveries in the pathogenesis of cutaneous diseases and the formulation of new treatments.

## Author contributions

Conceptualisation: B.L., F.M.W.; methodology: B.L., M.T., A.W.B., Z.J.G., D.V.S., C.P.; validation: B.L., A.W.B.; investigation: B.L., A.W.B., C.P., V.A.N., S.Z.; resources: M.T., C.P.; writing – original draft preparation: B.L., F.M.W.; writing – review and editing: all; visualisation: BL; funding acquisition: F.M.W., Z.J.G., D.V.S.

## Declaration of Competing Interest

The authors declare that they have no known competing financial interests or personal relationships that could have appeared to influence the work reported in this paper.

## Appendix Supplementary materials

Image, application 1
