# Supplementary material for: A reductionist approach to determine the effect of cell-cell contact on human epidermal stem cell differentiation
Source: Acta Biomater. 2022 Sep 15;150:265–76. doi: 10.1016/j.actbio.2022.07.054 (PMC9810539; doi:10.1016/j.actbio.2022.07.054)
Supplement: Supplementary file 1 [file mmc1.docx]

**Supporting information**

**A reductionist approach to determine the effect of cell-cell contact on human epidermal stem cell differentiation**

Blaise Louis^1^, Mukul Tewary^1^, Andrew W. Bremer^2,3^, Christina Philippeos^1^, Victor A. Negri^1^, Sebastiaan Zijl^1^, Zev J. Gartner^3^, David V. Schaffer^2^ & Fiona M. Watt^1*^

^1^Centre for Stem Cells and Regenerative Medicine, King’s College London, Guy’s Hospital, London, SE1 9RT, UK

^2^University of California, Berkeley, 278 Stanley Hall, Berkeley, CA 94720-3220, USA

^3^Department of Pharmaceutical Chemistry, University of California at San Francisco, 600 16th St, Rm N512E, UCSF Box 2280, San Francisco, CA 94158, USA

*Corresponding author: [fiona.watt@kcl.ac.uk](mailto:fiona.watt@kcl.ac.uk)

Pages: 12

Number of tables: 3

Number of figures: 8

| 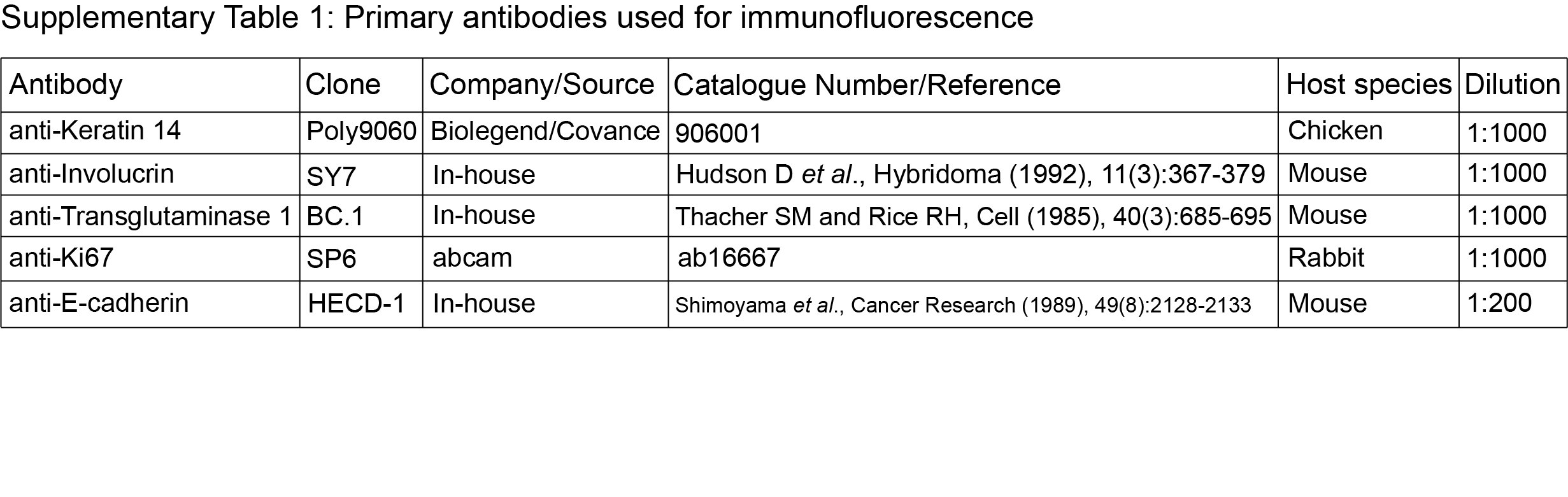 |
| --- |
| **Supplementary Table 1.** Primary antibodies used for immunofluorescence |

|  |
| --- |
| **Supplementary Table 2.** Oligonucleotide sequences and lipid-modifications |

|  |
| --- |
| **Supplementary Table 3.** qPCR primer sequences |

| 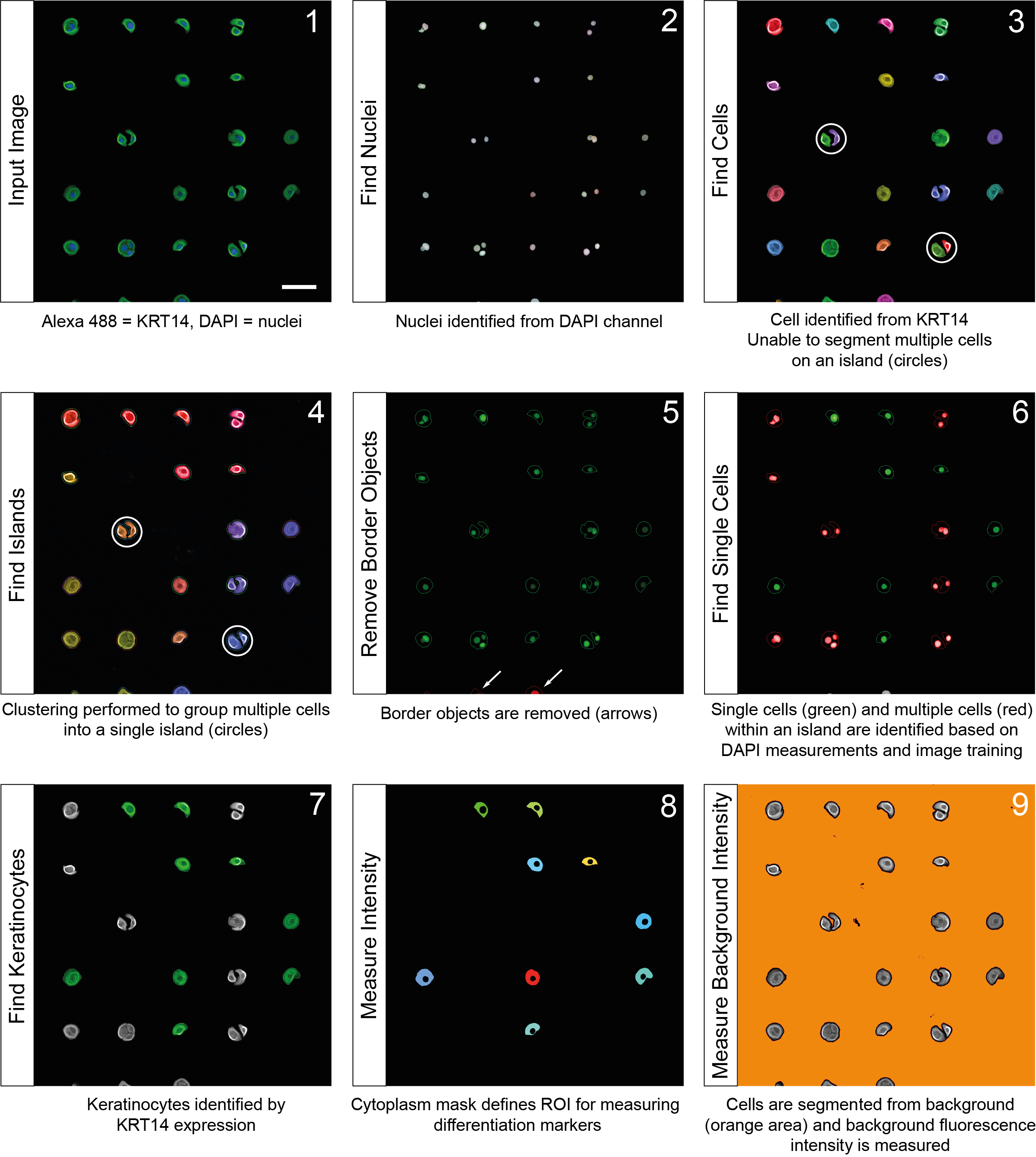 |
| --- |
| **Supplementary Figure 1.** Overview of the automated imaging analysis pipeline for micropatterned plates. From the (1) input image, (2) nuclei were identified from the DAPI channel. (3) Cells were identified by keratin 14 labelling. (4) Clustering was performed to group multiple cells in immediate proximity within each island, therefore identifying island regions (white circles) and (5) border objects were removed (white arrows). (6) Islands containing single or multiple cells were identified and (7) single cells were classified as keratinocytes based on keratin 14 expression. (8) ROI (cytoplasmic or nuclear) were applied and marker intensities were measured. (9) Cells were segmented from the image background, and the background intensity was measured. Scale bar, 100 μm. |

| 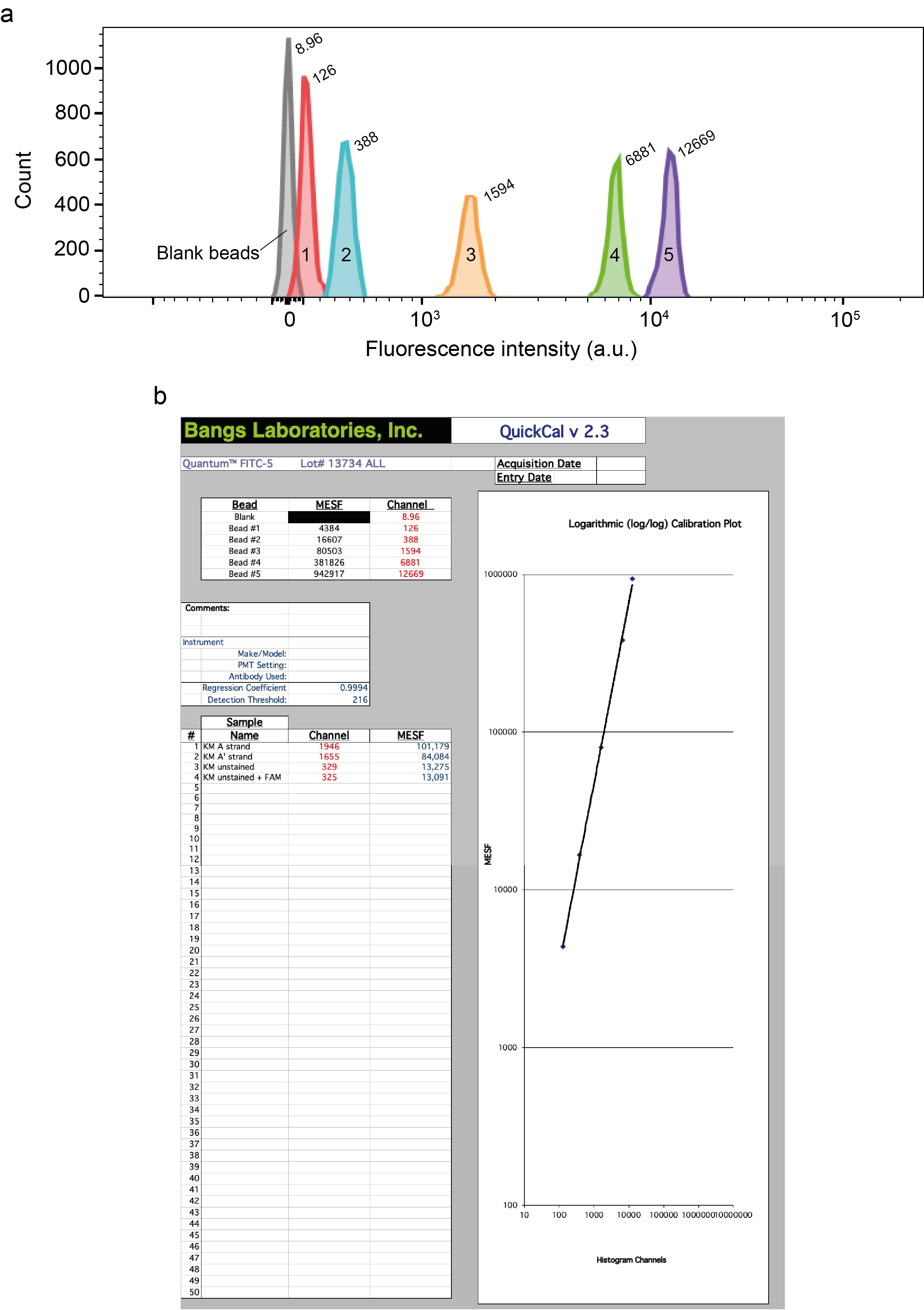 |
| --- |
| **Supplementary Figure 2.** Assessment of DNA incorporation into cells. (**a**) The median fluorescence intensities of Quantum MESF beads were determined by flow cytometry analysis. (**b**) MESF values (strands per cell) were obtained by inputting the median fluorescence intensities of the beads and labelled cells into the QuickCal v. 2.3 data analysis program. |
| 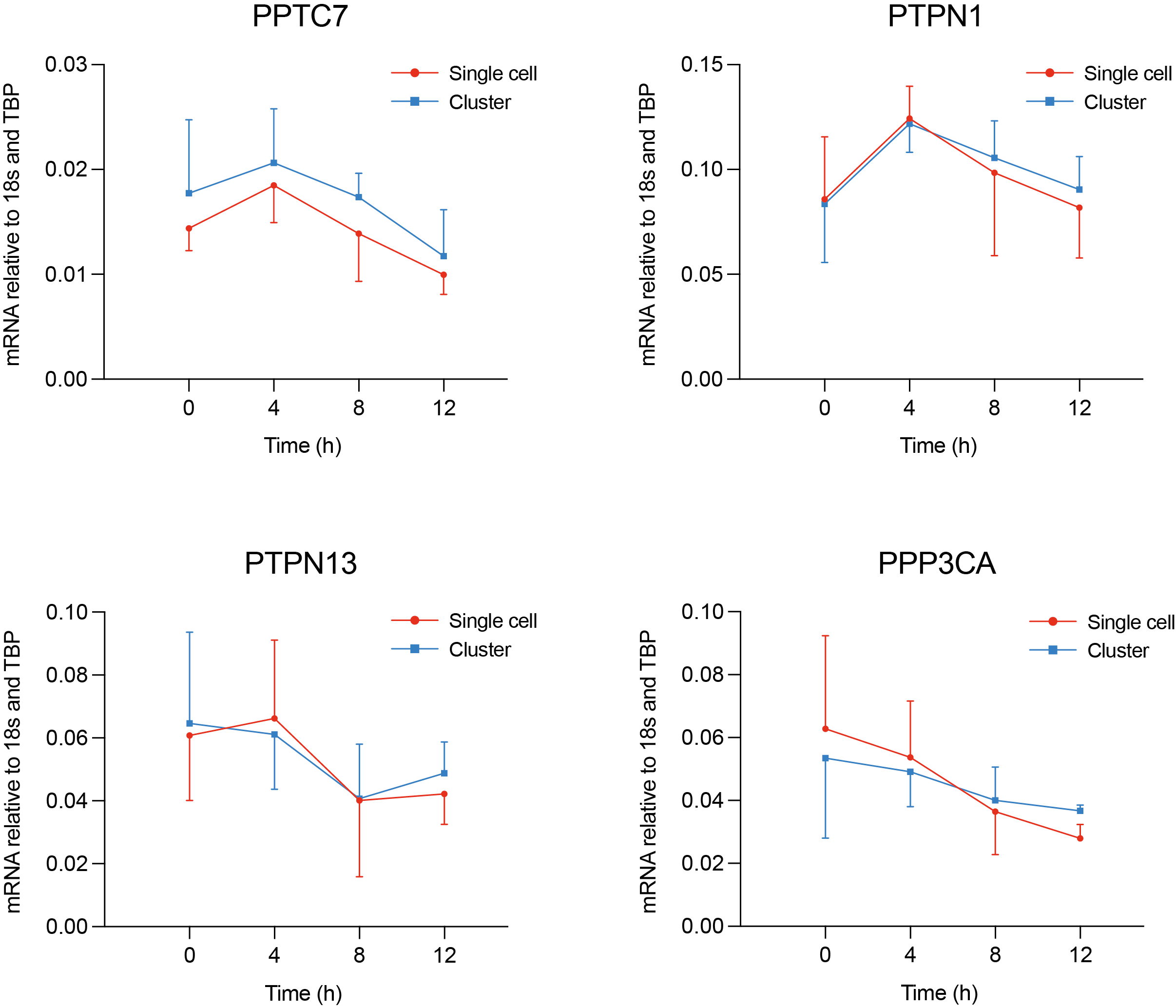 |
| **Supplementary Figure 3.** Expression of pro-commitment phosphatases in single and clustered cells in suspension. qPCR quantification of PPTC7, PTPN1, PTPN13 and PPP3CA mRNA levels relative to 18S and TBP for single cells (red) and clustered cells (blue). Data points represent the mean of n = 3 independent experiments and error bars represent SD. |

| 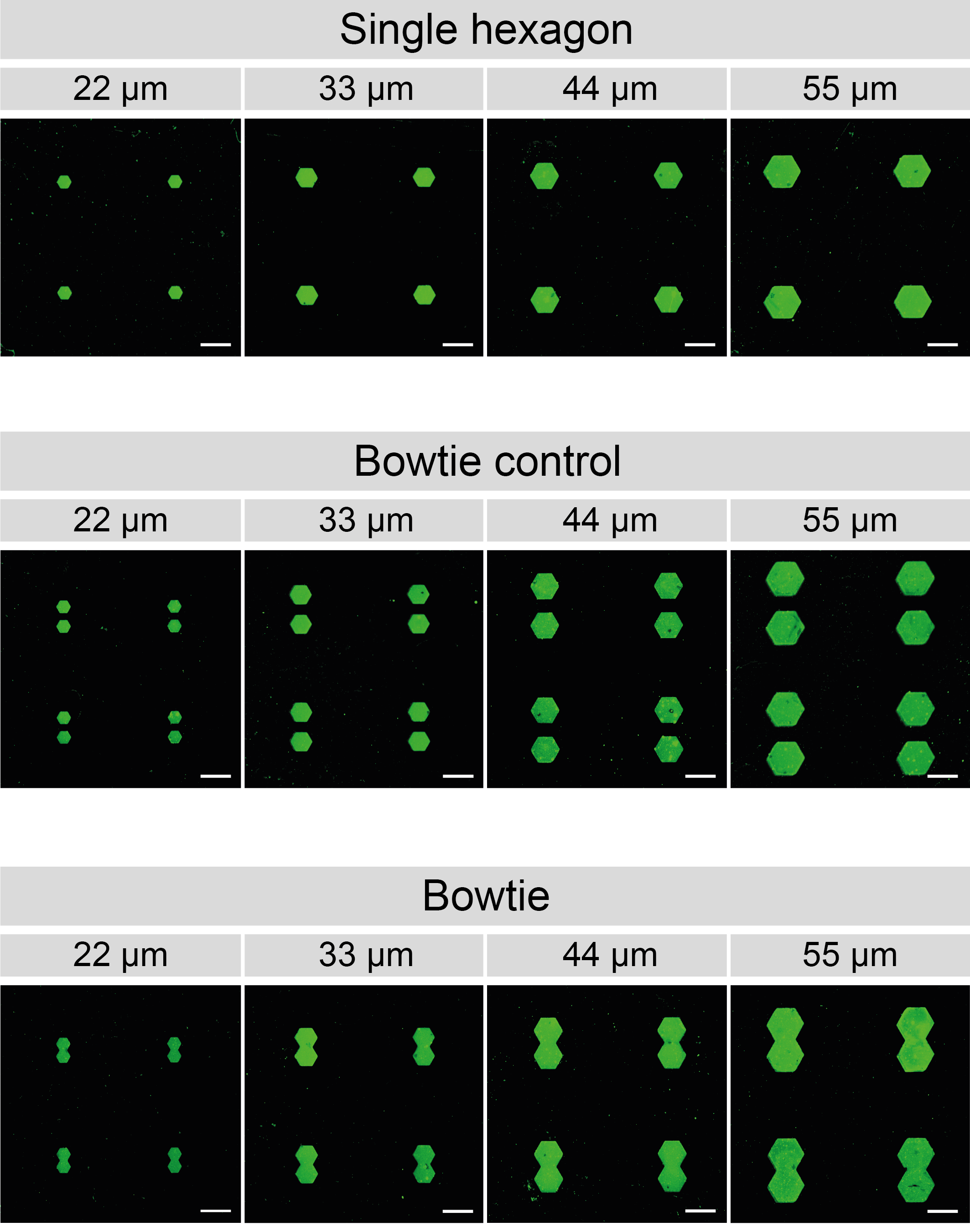 |
| --- |
| **Supplementary Figure 4.** Hexagonal bowtie-shaped micropatterned islands for modelling cell-cell contact. Representative immunofluorescence microscopy images of single and bowtie-shaped hexagonal islands coated with Alexa Fluor 488-conjugated fibrinogen. Areas of single hexagons and hexagons in a bowtie are equivalent to circular islands with diameters of 22, 33, 44 and 55 μm. Scale bar, 100 μm. |

| 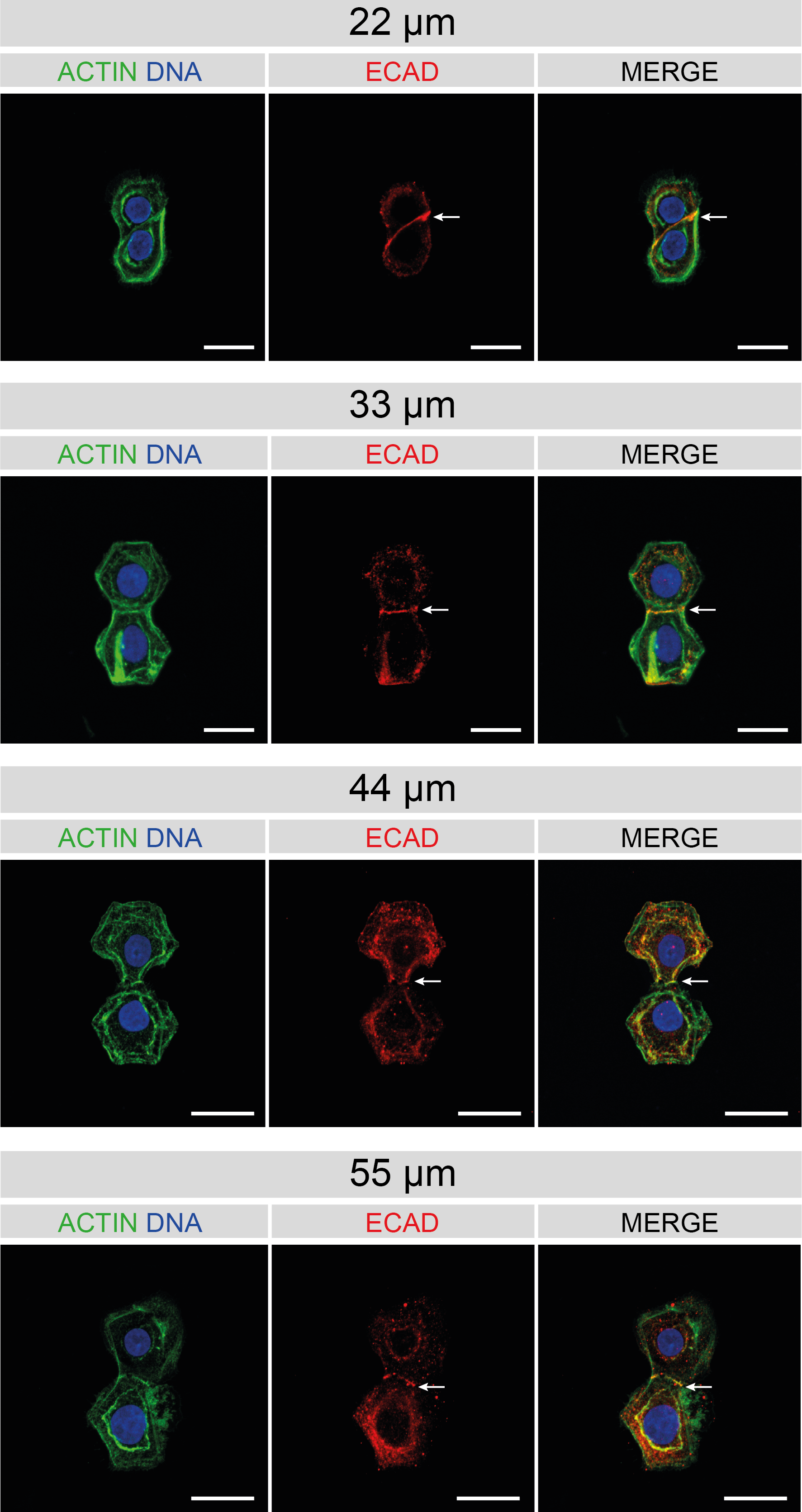 |
| --- |
| **Supplementary Figure 5.** Keratinocyte pairs express E-cadherin at cell junctions after 24 h on bowtie-shaped micropatterned islands. Representative immunofluorescence images of keratinocytes on 22, 33, 44 and 55 μm bowtie-shaped islands at 24 h after seeding. Cells were labelled for actin (green) and E-cadherin (ECAD, red), with DAPI (blue) as a nuclear counterstain. White arrows indicate E-cadherin localised to cell-cell junctions. Scale bars, 20 μm. |

| 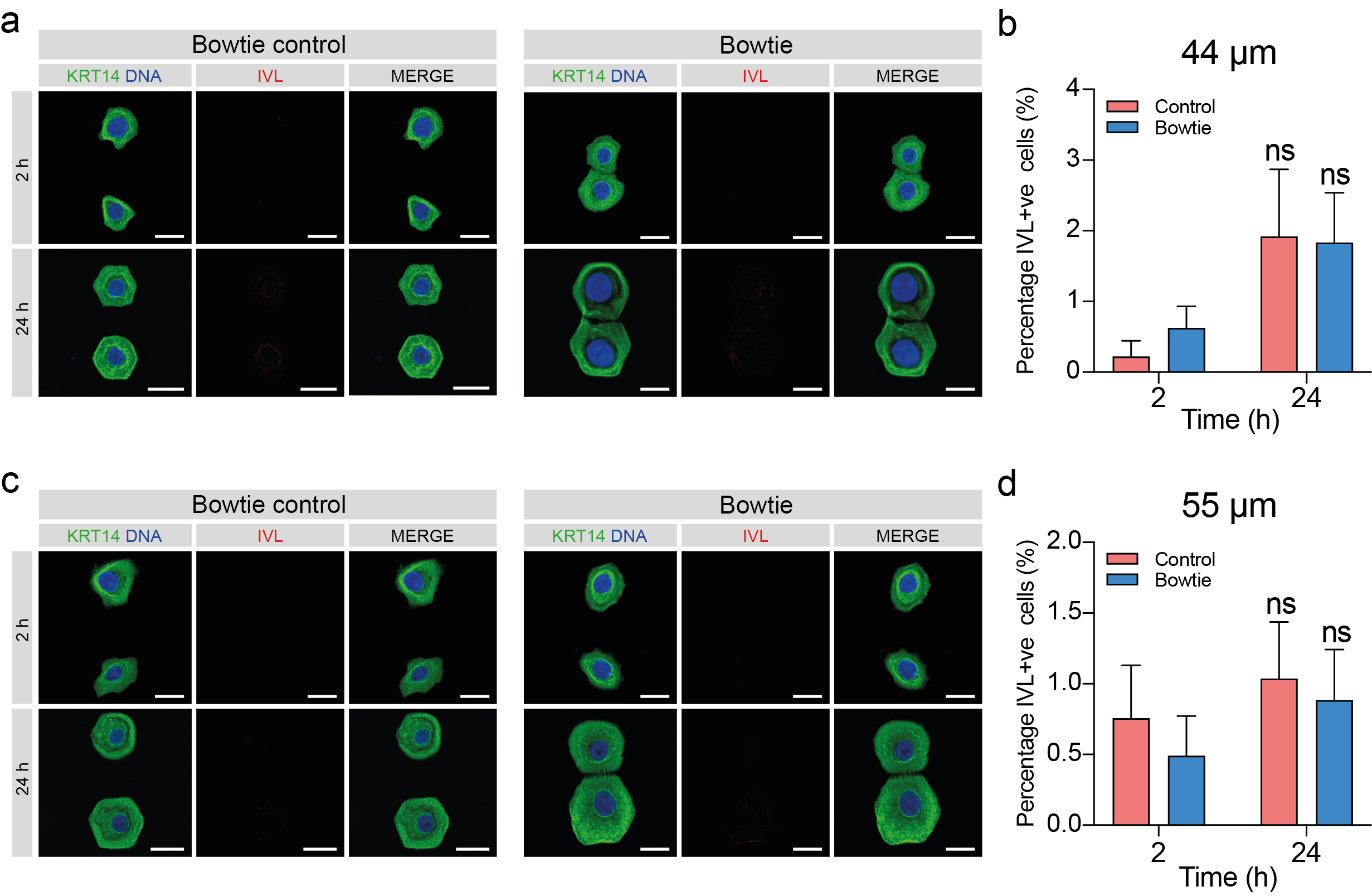 |
| --- |
| **Supplementary Figure 6.** Involucrin expression in keratinocyte pairs on large bowtie-shaped micropatterned islands. (**a**, **c**) Representative immunofluorescence images of keratinocytes on (**a**) 44 μm and (**c**) 55 μm bowtie-shaped islands at 2 and 24 h after seeding. Cells were labelled for keratin 14 (KRT14, green) and involucrin (IVL, red), with DAPI (blue) as a nuclear counterstain. Scale bars, 20 μm. (**b**, **d**) Quantification of the percentage of involucrin-positive cells for (**b**) 44 μm bowtie-shaped islands in the experiment shown in **a**, and (**d**) 55 μm bowtie-shaped islands in the experiment shown in **b**. Data shown are from n = 4 independent experiments. Bars represent mean ± SEM. ns = not significant (p > 0.05) as determined by two-way ANOVA with Šidák’s multiple comparisons test. |

| 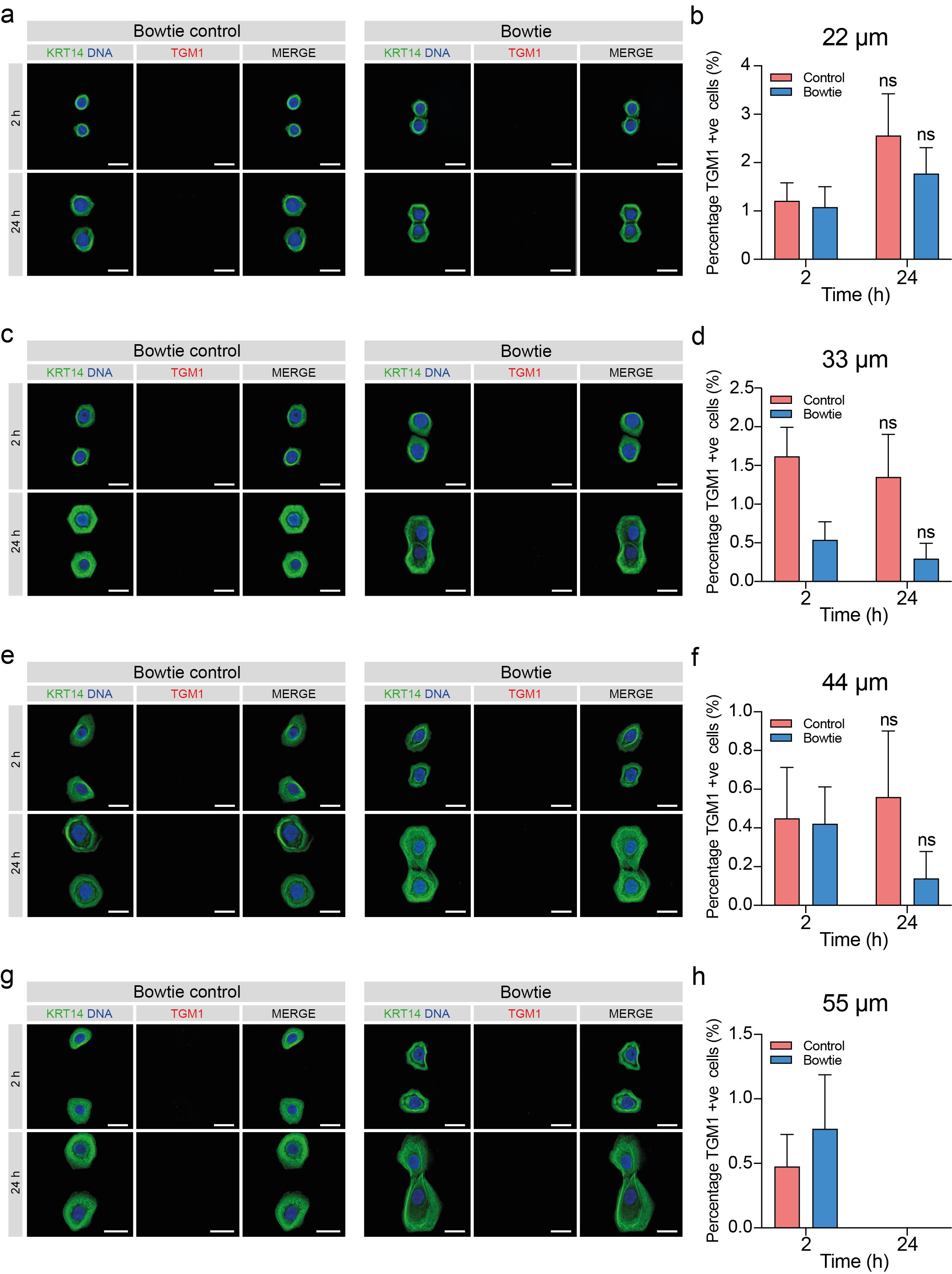 |
| --- |
| **Supplementary Figure 7.** Transglutaminase 1 expression in keratinocyte pairs on bowtie-shaped islands. (**a**, **c**, **e**, **g**) Representative immunofluorescence images of keratinocytes on bowtie-shaped islands at 2 and 24 h after seeding. Cells were labelled for keratin 14 (KRT14, green) and transglutaminase 1 (TGM1, red), with DAPI (blue) as a nuclear counterstain. Scale bars, 20 μm. (**b**, **d**, **f**, **h**) Quantification of the percentage of transglutaminase 1 positive cells in the experiments shown in **a**, **c**, **e** and **g**. Data shown are from n = 3 independent experiments. Bars represent mean ± SEM. ns = not significant (p > 0.05) as determined by two-way ANOVA with Šidák’s multiple comparisons test. |
| 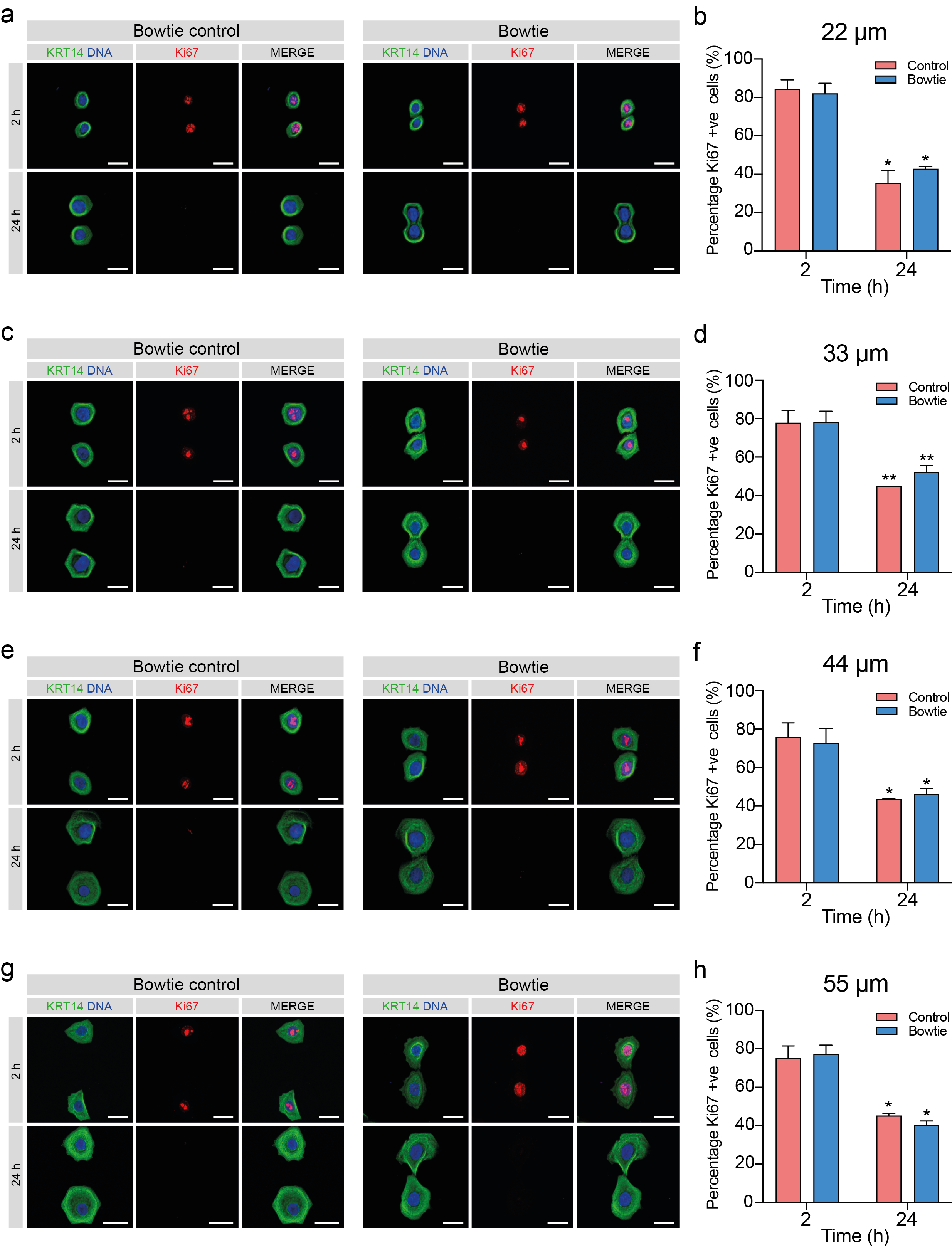 |
| **Supplementary Figure 8.** Ki67 expression in keratinocyte pairs on bowtie-shaped islands. (**a**, **c**, **e**, **g**) Representative immunofluorescence images of keratinocytes on bowtie-shaped islands at 2 and 24 h after seeding. Cells were labelled for keratin 14 (KRT14, green) and Ki67 (red), with DAPI (blue) as a nuclear counterstain. Scale bars, 20 μm. (**b**, **d**, **f**, **h**) Quantification of the percentage of Ki67 positive cells in the experiments shown in **a**, **c**, **e** and **g**. Data shown are from n = 2 independent experiments. Bars represent mean ± SEM. *p < 0.05 and **p < 0.01 as determined by two-way ANOVA with Šidák’s multiple comparisons test. |
